# A case of abiraterone acetate withdrawal syndrome after initiation of upfront abiraterone therapy for high‐risk prostate cancer

**DOI:** 10.1002/iju5.12604

**Published:** 2023-07-21

**Authors:** Masaru Tani, Yujiro Hayashi, Airi Miki, Teppei Wakita, Yuki Horibe, Yoichi Kakuta, Koichi Tsutahara, Tetsuya Takao

**Affiliations:** ^1^ Department of Urology Osaka General Medical Center Osaka Japan

**Keywords:** abiraterone, bone metastasis, hormone‐sensitive prostate cancer

## Abstract

**Introduction:**

Transient decrease in serum prostate‐specific antigen level can occur after abiraterone acetate withdrawal in male patient with metastatic castration‐resistant prostate cancer. Here, we report a case of abiraterone acetate withdrawal syndrome with transient prostate‐specific antigen decrease after progression to castration‐resistant disease while using upfront abiraterone therapy for high‐risk prostate cancer.

**Case presentation:**

A 73‐year‐old man with hormone‐sensitive high‐risk prostate cancer with multiple bone metastases (prostate‐specific antigen level, 294.109 ng/mL) received upfront abiraterone/prednisolone combination and androgen deprivation therapy. One year later, prostate‐specific antigen level decreased to 0.017 ng/mL (nadir) but it gradually rose by 15 months after treatment initiation. He was diagnosed as castration‐resistant and new bone metastases appeared. After abiraterone was discontinued, prostate‐specific antigen level decreased and stabilized at a low level for 5 months.

**Conclusion:**

Abiraterone acetate withdrawal syndrome was observed when hormone‐sensitive prostate cancer with upfront abiraterone therapy progressed to castration‐resistant prostate cancer.

**Abbreviations & Acronyms**
AA: abiraterone acetate
AAWS: abiraterone acetate withdrawal syndrome
AWS: antiandrogen withdrawal syndrome
CRPC: castration‐resistant prostate cancer
CT: computed tomography
HSPC: hormone‐sensitive prostate cancer
mHSPC: metastatic HSPC
MRI: magnetic resonance imaging
PSA: prostate‐specific antigen

**Keynote message**

AAWS has been reported during CRPC. This is the first case of AAWS after initiation of upfront AA therapy for high‐risk hormone‐sensitive prostate cancer.

## Introduction

In 2012, cessation of AA was first reported to be associated with AAWS in metastatic CRPC,^1^ but there have been no reports of AAWS after abiraterone treatment for HSPC. AAWS is characterized by a temporary decline in PSA after discontinuation of AA, which mimics AWS.^2^ We report the case of a patient with a decrease in PSA after discontinuation of AA when mHSPC with upfront abiraterone treatment progressed to CRPC.

## Case presentation

**Fig. 1:**
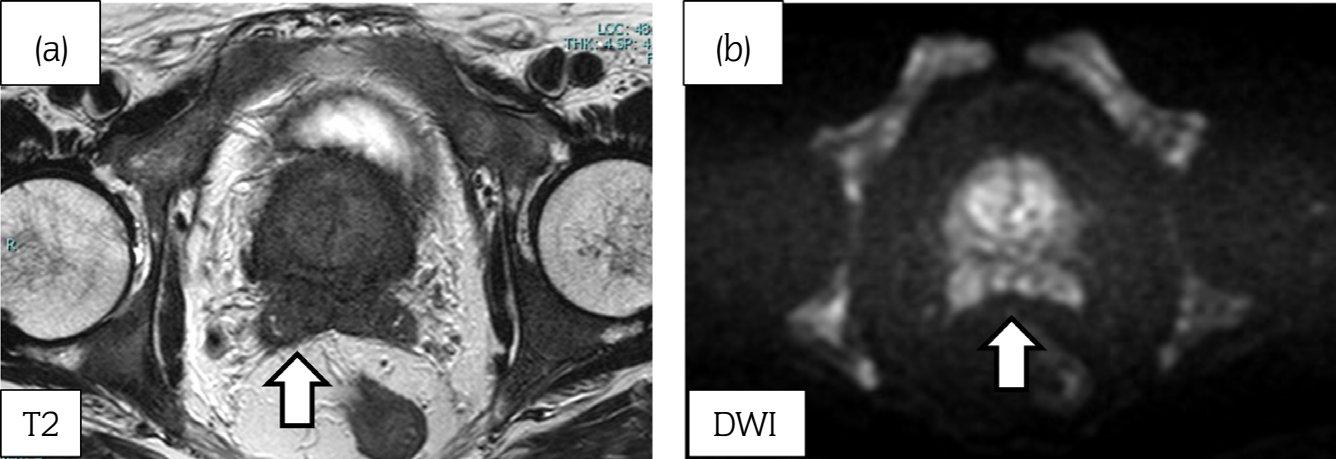
MRI of the prostate showed an indistinct hypointensity on T2‐weighted images (a) with a focal area of diffusion restriction. (b).

**Fig. 2:**
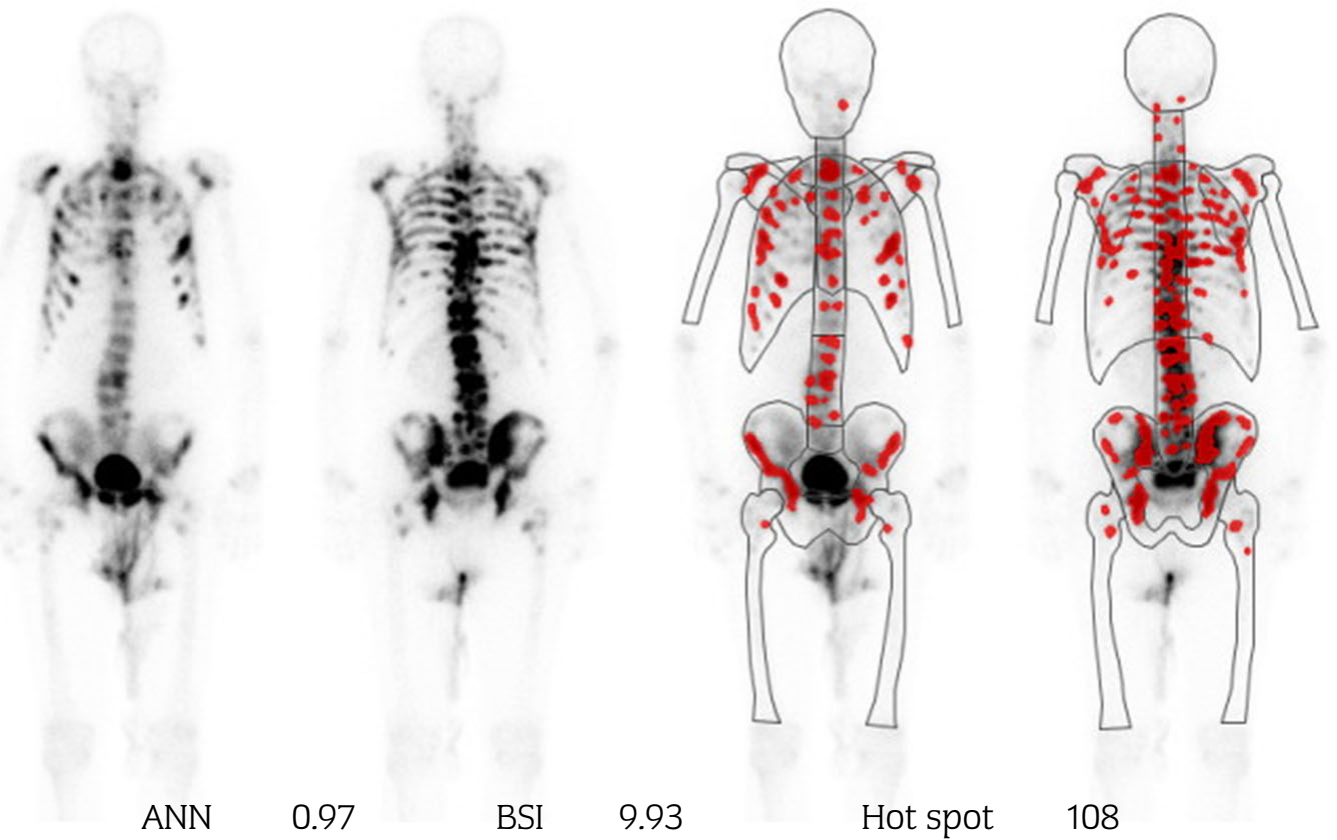
Bone scintigraphy at the time of diagnosis showed a high volume of multiple bone metastases.

A 73‐year‐old man with lumbago was referred to our institution in June 2018 for further detailed evaluation of serum PSA elevation, which had reached 294.109 ng/mL. We performed transrectal prostate needle biopsy. Prostatic adenocarcinoma and Gleason grade of 4 + 5 was shown by pathological examination. MRI revealed a massive prostate tumor, which directly invaded the anterior wall of rectum, seminal vesicles, and bladder (Fig. 1a,b). CT showed metastasis to the pelvic lymph nodes. Bone scintigraphy revealed multiple bone metastasis including lumbar spine (Fig. 2). He was diagnosed as having prostate cancer, clinical stage T4N1M1b. The patient had a Gleason score of 8 or higher and ≥3 bone metastases as high‐risk prognostic factors. Upfront AA therapy was initiated for mHSPC.

**Fig. 3:**
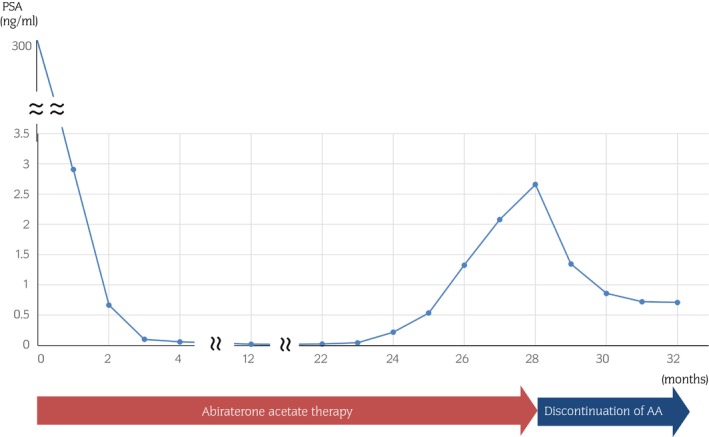
Patient's clinical treatment course. The graph shows the trend in changes of the patient's PSA levels.

Immediately after starting upfront combination therapy with AA, his PSA levels decreased rapidly and stabilized at a low level. At 24 months later, PSA levels had risen slightly, and at 27 months, his PSA levels were above 2 ng/mL. At this point, we decided to discontinue AA but continued to administer prednisolone while considering the possibility of introducing docetaxel if his PSA level rose further. However, his PSA level decreased and maintained a decreasing trend for 5 months (Fig. 3). During AAWS period with low PSA level, every 3 months CT scans showed no progression of bone lesions and no new metastatic sites including bone, lymph node, and other organs. We considered the patient to have AAWS when mHSPC with abiraterone treatment progressed to CRPC.

## Discussion

In recent years, evidence of the efficacy of so‐called upfront therapies such as AA, enzalutamide, and apalutamide has accumulated and become widely available for mHSPC. AA inhibits the androgen biosynthesis through the selective and irreversible inhibiting the CYP17 enzyme, which is essential for the androgen and estrogen synthesis.^3^, ^4^ AAWS is characterized by a temporary decline in PSA after discontinuation of AA, which mimics AWS.^2^ Cessation of AA was first reported in 2012 to be associated with AAWS in metastatic CRPC.^1^ However, to our knowledge, there has not been a single report of AAWS in patients who started AA for treatment of mHSPC. In the present case, the development of AAWS after discontinuation of AA due to the patient's elevated PSA level delayed the induction of chemotherapy with docetaxel therapy. In previous reports, in 15%–30% of the patients under treatment with AA, AAWS was observed.^5^ A report by Caffo *et al*. observed 26 patients with CRPC on AA after docetaxel chemotherapy, and when AA was discontinued due to elevated PSA, three patients had PSA reductions of 50% or more and one patient had resolution of liver metastases. The possibility of AAWS significantly affecting overall survival has also been suggested.^6^ A retrospective study of 70 cases of CRPC to examine predictors of AAWS expression was reported by Almendros *et al*.^5^ Among the 70 patients who discontinued AA due to elevated PSA, 11 patients (15.7%) developed AAWS. The predictive factors of AAWS are considered to be (1) a PSA level of 30 ng/mL or higher at the time of AA induction, (2) ISUP Gleason grade group 4 or higher, and (3) stage 4 at diagnosis. Although the pathophysiological mechanism is not fully understood, several hypotheses have been proposed. The receptor of mutant androgen is activated by alternative ligands, and ligand‐binding domains may contribute to AAWS.^7^, ^8^ Androgen receptor mutations in the ligand‐binding domain such as T878A or H874Y are speculated to be responsible for reduced stimulatory effects of antiandrogens on prostate cancer.^9^ AA administration slightly increases serum and tissue progesterone levels, and the elevated progesterone activates several mutant androgen receptors, including T878A, which are involved in the progression of prostate cancer.^10^ Therefore, we speculate that AA discontinuation decreases progesterone levels and mutant androgen receptor activation, resulting in the expression of AAWS. It has been reported that AWS that develops upon discontinuation of bicalutamide or flutamide is due to the drug acting as an agonist for the mutated AR.^11^ There are a few reports of withdrawal syndrome after discontinuation of enzalutamide, and 5 of 72 mCRPC patients who discontinued enzalutamide had a decrease in PSA. The median duration of response was only 6 weeks,^12^ and the mechanism of action has not been fully investigated. There are no reports of withdrawal syndrome after discontinuation of apalutamide. AAWS occurring after discontinuation of abiraterone has been reported more frequently than with other new hormonal agents, and the difference in the pharmacological mechanism may be associated with the difference in frequency of occurrence. We speculate that AA discontinuation caused a downregulatory role at multiple sites in the AR signaling pathway, resulting in the development of AAWS. It has also been reported that concomitant prednisolone also alters androgen synthesis, but Kato *et al* reported that the frequency of AAWS was not associated with prednisolone use, suggesting that the AAWS might be mainly associated with the discontinuation of AA.

We described a case of AAWS in a patient being treated with upfront AA therapy for mHSPC. When a patient with mHSPC treated by upfront AA is found to have an elevated PSA, the patient should not be immediately shifted to the next treatment. Possible pharmacological mechanism of discontinuation of AA is thought to downregulate AR signaling, AAWS is expected to occur promptly after AA discontinuation, as in this case. If the patient's condition permits, we think discontinuation of AA administration for about a month with PSA follow‐up would be clinically acceptable, and if PSA levels do not decrease after AA discontinuation, clinician should move forward the next treatment. The advantages of confirming AAWS include the ability to increase the patient's quality of life by monitoring the patient's progress without administering drugs, and it may also be useful in terms of medical economics.

In conclusion, the frequency of AAWS is likely to be higher and more useful than we had expected, further data accumulation is needed to select appropriate cases in which AAWS should be confirmed and to elucidate the mechanism.

## Author contributions

Masaru Tani: Conceptualization; data curation; formal analysis; investigation; visualization; writing – original draft; writing – review and editing. Yujiro Hayashi: Conceptualization; supervision; writing – original draft; writing – review and editing. Airi Miki: Supervision. Teppei Wakita: Supervision. Yuki Horibe: Supervision. Yoichi Kakuta: Conceptualization; supervision. Koichi Tsutahara: Conceptualization; supervision. Tetsuya Takao: Conceptualization; supervision; writing – review and editing.

## Conflict of interest

The authors declare no conflict of interest.

## Approval of the research protocol by an Institutional Reviewer Board

Not applicable.

## Informed consent

Informed consent was obtained from patient.

## Registry and the Registration No. of the study/trial

Not applicable.
